# Genetic Modifiers of Mendelian Monogenic Collagen IV Nephropathies in Humans and Mice

**DOI:** 10.3390/genes14091686

**Published:** 2023-08-25

**Authors:** Constantinos Deltas, Gregory Papagregoriou, Stavroula F. Louka, Apostolos Malatras, Frances Flinter, Daniel P. Gale, Susie Gear, Oliver Gross, Julia Hoefele, Rachel Lennon, Jeffrey H. Miner, Alessandra Renieri, Judy Savige, A. Neil Turner

**Affiliations:** 1 School of Medicine, University of Cyprus, Nicosia 2109, Cyprus; 2 biobank.cy Center of Excellence in Biobanking and Biomedical Research, University of Cyprus, Nicosia 2109, Cyprus; 3 Clinical Genetics Department, Guy’s & St Thomas’ NHS Foundation Trust, London SE1 9RT, UK; 4 Department of Renal Medicine, University College London, London NW3 2PF, UK; 5 Alport UK, Tetbury GL8 0AW, UK; 6 Clinic for Nephrology and Rheumatology, University Medicine Goettingen, 37075 Goettingen, Germany; 7 Institute of Human Genetics, Klinikum Rechts der Isar, School of Medicine & Health, Technical University Munich, 81675 Munich, Germany; 8 Wellcome Centre for Cell-Matrix Research, University of Manchester, Manchester M13 9WU, UK; 9 Division of Nephrology, Department of Medicine, Washington University School of Medicine, St. Louis, MO 63110, USA; 10 Medical Genetics, University of Siena, 53100 Siena, Italy; 11 Med Biotech Hub and Competence Center, Department of Medical Biotechnologies, University of Siena, 53100 Siena, Italy; 12 Genetica Medica, Azienda Ospedaliero-Universitaria Senese, 53100 Siena, Italy; 13 Department of Medicine (Melbourne Health and Northern Health), The University of Melbourne, Parkville, VIC 3052, Australia; 14 Renal Medicine, Royal Infirmary, University of Edinburgh, Edinburgh EH16 4UX, UK

**Keywords:** Alport syndrome, thin basement membrane nephropathy, focal segmental glomerulosclerosis, glomerular diseases, *COL4* nephropathies, genetic modifiers

## Abstract

Familial hematuria is a clinical sign of a genetically heterogeneous group of conditions, accompanied by broad inter- and intrafamilial variable expressivity. The most frequent condition is caused by pathogenic (or likely pathogenic) variants in the collagen-IV genes, *COL4A3/A4/A5*. Pathogenic variants in *COL4A5* are responsible for the severe X-linked glomerulopathy, Alport syndrome (AS), while homozygous or compound heterozygous variants in the *COL4A3* or the *COL4A4* gene cause autosomal recessive AS. AS usually leads to progressive kidney failure before the age of 40-years when left untreated. People who inherit heterozygous *COL4A3*/*A4* variants are at-risk of a slowly progressive form of the disease, starting with microscopic hematuria in early childhood, developing Alport spectrum nephropathy. Sometimes, they are diagnosed with benign familial hematuria, and sometimes with autosomal dominant AS. At diagnosis, they often show thin basement membrane nephropathy, reflecting the uniform thin glomerular basement membrane lesion, inherited as an autosomal dominant condition. On a long follow-up, most patients will retain normal or mildly affected kidney function, while a substantial proportion will develop chronic kidney disease (CKD), even kidney failure at an average age of 55-years. A question that remains unanswered is how to distinguish those patients with AS or with heterozygous *COL4A3/A4* variants who will manifest a more aggressive kidney function decline, requiring prompt medical intervention. The hypothesis that a subgroup of patients coinherit additional genetic modifiers that exacerbate their clinical course has been investigated by several researchers. Here, we review all publications that describe the potential role of candidate genetic modifiers in patients and include a summary of studies in AS mouse models.

## 1. Introduction

Familial microscopic hematuria of a glomerular origin is the presenting feature in several inherited monogenic conditions, the most frequent of which is a spectrum of type IV collagen nephropathy including Alport syndrome (AS). AS follows X-linked inheritance due to pathogenic, disease-causing variants in the *COL4A5* gene, or autosomal recessive inheritance (ARAS) caused by homozygous or compound heterozygous variants in either the *COL4A3* or the *COL4A4* gene [1]. According to guidelines, the diagnostic criteria for AS include persistent microscopic hematuria, which is supported by the reporting of a positive family history and characteristic extra-renal clinical features [2]. On kidney biopsy imaging, pathognomonic features of AS include variable thinning and thickening of the glomerular basement membrane (GBM), splitting and lamellation. When proteinuria develops, there is podocyte foot processes effacement [3,4].

With regards to the *COL4A3* and *COL4A4* disease-causing variants, the gene dose makes a difference on the disease development and spectrum of features. While patients with homozygous or compound heterozygous variants will usually manifest typical ARAS, those inheriting pathogenic variants in a heterozygous state sometimes present with a thin glomerular basement membrane lesion. This uniform thinning of the GBM leads to microscopic hematuria, due to leaking of red blood cells into the urine. This was formerly named, and is still used by some, as thin basement membrane nephropathy (TBMN). Also, a thin GBM lesion and microscopic hematuria may be caused by mutations in other genes [5,6,7].

At a very young age, persistent microscopic hematuria associated with GBM thinning cannot be differentiated from typical AS, and genetic testing emerges as the gold standard for establishing the etiology, in many cases, precluding the need for a kidney biopsy if not performed already [8,9]. At later ages, in addition to kidney function impairment, most patients with AS also develop extra-renal features, including hearing loss and ocular abnormalities (anterior lenticonus, maculopathy, retinopathy), findings which are very rare in patients with heterozygous *COL4A3* or *COL4A4* disease causing variants [10].

In patients with heterozygous pathogenic variants in the *COL4A3/A4*, while most frequently there is only uniform thinning of the GBM, some patients develop renal features reminiscent of typical AS, but with a much later age for the onset of kidney failure [10]. A central question that remains unexplained is why some patients do worse than others, even within the same families.

During the recent International Workshops on Alport Syndrome held in Siena, Italy, from 22 to 26 October 2019, and online, from 30 November to 4 December 2021, members of the International Alport Syndrome Alliance discussed the issue of clinical heterogeneity in patients who carry heterozygous pathogenic variants in the *COL4A3* or *COL4A4* genes and present with Alport spectrum disease, while others present with later-onset phenotypes due to a thinner than normal GBM. Amongst experts, there is a debate, as several members of the international nephrology community prefer to diagnose these heterozygous patients with TBMN, others with autosomal dominant Alport syndrome (ADAS) [11]. This manuscript does not intend to address the naming of this condition. In the absence of a more appropriate terminology, the fact remains that although most patients live with normal or minimally affected kidney function for life, a variable and largely unpredictable subset of them progress to severe CKD, including kidney failure, after developing focal and segmental glomerulosclerosis on biopsy. In fact, on several occasions, these patients are misdiagnosed with primary focal and segmental glomerulosclerosis and treated with immunosuppressive medications, until genetic testing enables reclassification, and they are treated with renin, angiotensin aldosterone system (RAAS) inhibitors [12,13,14,15].

In patients with heterozygous *COL4A3/A4* pathogenic variants, while it is agreed that the actual variant type and location determine to some extent the spectrum of clinical manifestations, it is widely accepted that other coinherited genetic variants may modify the phenotype. This is particularly evident in families where multiple affected members inherit the same heterozygous DNA variant (usually a glycine substitution or other variant, e.g., indels), but intrafamilial heterogeneity is evident by a variable degree of proteinuria in different members and disparate ages at the onset of kidney failure, sometimes differing by 10, or even 20, years. Ultrastructural findings on kidney biopsy images of electron microscopy can also attest to this variable expressivity, as few patients will manifest defects beyond the typical universal thinning of the GBM and only a small percentage will develop hearing problems, which may be coincidental (hearing loss in later life is very common in the general population). Matthaiou et al. [10] showed previously, in a systematic review of 777 patients from 258 families of mostly diverse Caucasian origin with segregating heterozygous *COL4A3/A4* defects, that the age at onset of kidney failure ranged between 21 and 84 years (mean 52.8). Kidney failure occurred in 15% of patients (104/687), hearing impairment in 16% (101/649) and ocular abnormalities in only 3% (16/525) (see Table 4 of ref. [10]). In the same study, the authors showed that patients harboring missense variants (mostly glycine substitutions) had an older age at the onset of kidney failure compared to those harboring other categories of variants that result in premature termination of translation and a truncated protein (deletions, duplications, frameshift, splice-site mutations). 

Similar observations regarding clinical heterogeneity have been made in patients who inherit pathogenic variants in the *COL4A5* gene, responsible for the X-linked form of AS. This is documented through genotype–phenotype correlations and include hypomorphic variants such as the *COL4A5*:p.Gly624Asp, which is most frequently, but not always, accompanied by a milder clinical picture [16,17]. The term “hypomorphic” was coined by the 1946 Nobel Prize winner Hermann J. Muller (1890–1967). It refers to DNA variants which, although they affect either the protein function or the level of gene expression, there is still residual activity maintaining a milder phenotype.

The kidney phenotype can be improved by nephroprotective therapy targeting the Renin-Angiotensin-Aldosterone System (RAAS); the RAAS-blockade slows down the progression to macro-albuminuria and delays the onset of kidney failure [18,19]. Also, recent data point to a genotype-response-to-therapy correlation; missense variants in the type-IV collagen genes lead to a better response to the RAAS-blockade compared to nonsense-variants in X-linked as well as in autosomal recessive AS [20]. In conclusion, knowing the exact causative genotype in type IV collagen diseases not only helps to improve family counselling in terms of the prognosis and risk of early kidney failure, but also enables the clinician to make a more informed decision regarding early treatment with nephroprotective therapy, aimed to delay the onset of kidney failure and offer a better quality of life [21].

To account for this extensive clinical heterogeneity, several authors have invoked the concept of genetic modifiers (GM). What is a genetic modifier? It may not be possible to agree on a universal definition. Some may consider that GMs operate as additional (one or more) molecular defects, which determine the final phenotype on the background of the primary *COL4A* pathogenic variant. In this sense, one could argue that the phenotype is the consequence of digenic inheritance in the sense that it “can be better explained” by invoking the contribution of two variants at different loci, genetically linked or unlinked, than each one on its own. A variation to this definition was offered by Deltas (2018): […a GM is a DNA variant that exerts an epistatic effect on the phenotype, which is invariably determined by a primary gene. The variable expressivity (clinical or phenotypic heterogeneity) may be confounded by the contribution of one or more GM. This phenotypic heterogeneity, which can be hugely variable, ranging from very benign to very severe and life threatening, is part of the spectrum of symptoms which pertain to the genotype/phenotype correlation focusing on the primary gene at fault] [22].

Admittedly, sometimes the distinction between digenic inheritance and GMs is blurred. Here, we present published examples of GMs identified by several groups in humans (Table 1) and a less extensive reporting on mice (Table 2).

## 2. Modifier Genes in Alport Spectrum Diseases

We refer to Alport spectrum diseases or Alport spectrum nephropathy as those conditions caused by heritable pathogenic variants in the collagen IV genes, also referred to as collagen IV nephropathies.

### 2.1. Slit Diaphragm NPHS2 and NPHS1 Gene Variants

Homozygotes or compound heterozygotes of disease-causing variants in *NPHS2* (podocin) have a hereditary form of steroid resistant nephrotic syndrome (SRNS), while carriers of heterozygous variants are healthy. Podocin is an important protein that interacts with nephrin at the podocyte slit diaphragm, which selectively allows molecules to be filtered [46,47]. Tonna et al. [23], on a small cohort of patients with TBMN, were the first to observe that 3/10 (30%) of patients with proteinuria ≥500 mg/day were heterozygous for the recessive NPHS2:*p*.Arg229Gln variant, whereas only two out of 46 (4%) of patients with less proteinuria were heterozygous (*p* < 0.05; chi-square test with Yate’s correction).

Voskarides et al. [24] reported on the presence of this variant in a larger mixed cohort that included 62 mildly affected patients with TBMN and CFHR5 nephropathy (44/18, respectively), and 85 severely affected (58/27, respectively). Note that *CFHR5* exon 2-3 duplication is endemic in Cyprus and is a hereditary form of complement C3 dysregulation, manifesting with microscopic hematuria since childhood. Although the gene involved has no functional relationship to collagen IV, and the pathology does not fall under the Alport spectrum diseases, the disease can follow a progressive course very similar to TBMN, for which reason patients with CFHR5 nephropathy were included by the authors in a common cohort of patients manifesting microscopic hematuria [48,49]. Patients were classified as severe if they had proteinuria higher than 500 mg/day, at any age, and as mild if they had no or low-grade proteinuria below 300 mg/day and were older than 50 years. The authors showed, with statistical significance, that the variant predisposes patients to proteinuria and CKD, even kidney failure (Mild vs Severe *p* = 0.01, and *p* = 0.043 when kinship was considered). 

Stefanou et al. provided additional evidence for the NPHS2:*p*.Arg229Gln variant’s potential modulating effect, along with another *NPHS2* variant, NPHS2:*p*.Glu237Gln. In tested families, both *NPHS2* variants segregated with severely affected patients and not with patients who had inherited the same *COL4A* gene primary variant but not the *NPHS2* variant, thereby enhancing its potential modifying role. Additionally, they reported functional studies in cell culture experiments, demonstrating impaired behavior regarding its localization and interacting partners [25]. Specifically, dual co-transfection of wild type (WT) and mutant podocin gene constructs, followed by immunofluorescence (IF) staining, suggested the altered co-localization of mutant homodimers. Co-transfection of WT podocin and nephrin, followed by IF experiments, showed normal membrane localization, while both podocin variants interfered with normal trafficking, demonstrating perinuclear staining. Immunoprecipitation experiments showed stronger binding of mutant podocin to WT podocin or nephrin.

Collectively, these data support the hypothesis that certain heterozygous podocin variants, which on their own would not lead to perceptible clinical effects, aggravate the phenotype when co-inherited on the background of heterozygous *COL4A3/A4* pathogenic variants, by predisposing to focal segmental glomerulosclerosis and kidney function decline. Obviously, this is the result of a slow and long process which applies a detrimental effect on the slit diaphragm, and thus impairing the glomerular filtration barrier. Also, it should be mentioned that these studies showed that only a fraction of the severely affected patients presented with the likely modifying variants tested, suggesting that other similar GMs might exist. 

Frese et al. [26] reported on three XLAS cases where the clinical condition of patients who inherited *COL4A5* pathogenic variants was aggravated, and they developed focal segmental glomerulosclerosis due to the co-inheritance of variants in slit diaphragm genes. In case-1, the affected boy (COL4A5:*p*.Trp1538X) co-inherited heterozygous NPHS1:*p*.Arg408Gln (MAF 4.87%) and developed unusually high-grade proteinuria (1300 mg/L) from the age of 2 years. Severe focal segmental glomerulosclerosis was shown on the biopsy, along with unusually gross abnormalities of the GBM and broadening of the podocyte foot processes, including a partial loss of the slit diaphragm.

In case-2, a heterozygous female patient (NM_0.33380.3_COL4A5:*c*.4821+3A>G; initially reported as: IVS49+3A>G), who co-inherited the pathogenic recessive podocin variant NPHS2:*p*.Arg229Gln, presented very early with severe disease, with biopsy-proven focal segmental glomerulosclerosis. She was transplanted at the very young age of 15 years, which is very unusual for female patients with a heterozygous variant. Her daughter had progressive proteinuria from the first year of life, and her biopsy at age 2 revealed advanced pathology on EM imaging.

In case-3, the primary causative variant in a female patient was the hypomorphic COL4A5:*p*.Gly624Asp, which is known to cause a milder and later onset phenotype even in males, leading to kidney failure on average 30 years later than in patients with other pathogenic variants. She also inherited the pathogenic recessive podocin variant NPHS2:*p*.Arg229Gln and presented with proteinuria at 27 years; a kidney biopsy demonstrated advanced focal and segmental glomerulosclerosis. She developed minimal high-frequency hearing loss and focal segmental glomerulosclerosis at 40 years of age and was transplanted at 51 years. Amongst five affected members, 4F/1M, the only female patient who had co-inherited the podocin variant, had the worst levels of proteinuria and albuminuria [26].

Daga et al. [27] reported a family with a father and son who had inherited COL4A4:*c*.4444del (*p*.Leu1482Trpfs*70), as the primary defect, plus another two variants: NPHS2:*p*.Arg229Gln and LAMA5:*p*.Thr774Ile. The authors, in their wording, diagnosed the patients with ADAS, the symptoms being microscopic hematuria but no proteinuria in either of them. A kidney biopsy in the proband revealed areas of thinning and thickening of the GBM, as well as podocyte foot processes retraction and simplification, and podocytes bulging. Both father and son had mild bilateral hypoacusia. The authors attributed this more penetrant ultrastructural presentation and hearing problems to the co-inheritance of the *COL4A4* with two additional variants. The case with the cosegregation of the *COL4A4* with the *LAMA5* variant could also be viewed as a case of digenic inheritance. 

### 2.2. NEPH3 (Filtrin, Kirrel2)

Perhaps the most extensive work concerning the potential role of a candidate GM in TBMN concerns the NEPH3:*p*.Val353Met variant (MAF around 3% in the general population), combining genetic and cell culture experimental work [28]. Variant Val353Met in filtrin was initially shown to have a negative association in a cohort of 103 TBMN patients classified as mild or severe, 78 of whom carried the founder pathogenic variant COL4A3:*p*.Gly1334Glu, 19 carried variant COL4A3:*p*.Gly871Cys and six carried variant COL4A4:c.3854delG (*p*.Asp1285Alafs*3), thus minimizing allelic heterogeneity. The risk predisposing effect was corroborated on a larger cohort with all samples pooled together (HEMATURIA cohort) of 524 subjects under the dominant model (*p* = 3.0 × 10^−3^, OR = 6.64 adjusting for gender/age; allelic association: *p* = 4.2 × 10^−3^ adjusting for patients’ kinships). This larger cohort included patients from Cyprus, Greece, the UK and Australia. Genotyping on other general population, where independent cohorts included 6531 subjects of the Framingham Heart Study and 4727 of the KORAF4 and SAPHIR cohorts, demonstrated the association of the homozygous 353M/M genotype with micro-albuminuria. A meta-analysis of all three cohorts (11,258 individuals) was highly significant (*p* = 1.3 × 10^−5^, OR = 7.46). Importantly, functional studies with co-immunoprecipitation experiments in HEK293T cells demonstrated that at a protein level, the 353Met variant interfered with Neph3 homodimerization and Neph3-Nephrin heterodimerization. Further experiments in undifferentiated podocytes stressed with tunicamycin showed that overexpression of the 353Met allele resulted in activation of the unfolded protein response (UPR), as evidenced by the elevation of the UPR markers BiP, IRE1a and p-elF2a. Notably, the sequence with the more common valine at position 353 of the protein, 353Val, shows absolute conservation on a large evolutionary scale, all the way from drosophila to mouse and human. Hence, three lines of evidence, evolutionary conservation, genetic association and cell culture functional assays, suggest that variant NEPH3:*p*.Val353Met is a hypomorphic variant with low or incomplete penetrance, in predisposing to glomerular albuminuria and adverse kidney function [28]. 

### 2.3. LAMA5 (Laminin α-5)

This encodes one of the α-subunits of trimeric laminin molecules, an abundant non-collagenous component of the GBM. Disease-causing variants in this gene have been found in patients with autosomal recessive nephrotic syndrome [50,51]. Voskarides et al. first reported on the putative GM role of variants in the *LAMA5* gene [29]. In a Cypriot family, there was variant COL4A5:*p*.Asp654Tyr, which is never reported in databases. With the use of ACMG criteria [52] and based on family segregation, it is classified as likely pathogenic. Two hypertensive brothers had hematuria and heavy nephrotic range proteinuria, as well as focal segmental glomerulosclerosis on biopsy. They both progressed to kidney failure in their fifties. EM was available in one of the two and showed a folded GBM with thickening in many areas. The podocytes were vacuolated and markedly fused. The two brothers had one daughter each, who presented with microscopic hematuria and proteinuria. Kidney biopsies at 24 and 25 years showed focal and segmental glomerulosclerosis. EM in one of the two daughters also showed GBM thinning alternating with thickening, with some podocytes showing segmental effacement. None of the patients had clear pathognomonic features of AS. All four patients had inherited the *COL4A5* variant and the rare variant LAMA5:*p*.Pro1243Leu. Its updated general population frequency is reported as 0.0038% (4/105,402, Franklin, ExAC database; https://franklin.genoox.com/clinical-db/home, accessed on 30 June 2023). The Franklin database classifies it as a VUS. A son in the family inherited only the *LAMA5* variant and at the age of 27 years is healthy, with no haematuria and a normal kidney function. Interestingly, the two brothers who developed kidney failure, while they do not have polycystic kidney disease, on an ultrasound, both have multiple cortical cysts, one since the age of 4 years. Kidney cysts in patients with TBMN have been reported before by several authors [53,54]. The role of *LAMA5* variants in health and disease has been shown in mice models, which also presented with a cystic phenotype, as well as in patients who presented with focal and segmental glomerulosclerosis [55]. A more recent report described four patients with congenital/infantile nephrotic syndrome who inherited truncating or missense recessive biallelic pathogenic or likely pathogenic variants in *LAMA5* [51].

Daga et al. [27] reported a family in which multiple members had presented with microscopic or macroscopic hematuria, proteinuria and sensorineural hearing loss bilaterally. Some progressed to kidney failure while a maternal grandfather had bilateral kidney cysts at around 45 years of age. Another younger member also showed one renal cyst. All severely affected patients had a COL4A4:*p*.Gly370Glu pathogenic variant and a hypomorphic variant LAMA5:*p*.His1717Tyr (Benign, 7.2204% in ExAC database). The authors consider that this represents digenic inheritance, although it is not clear whether the *LAMA5* variant would cause a phenotype on its own in a heterozygous state; it could equally be viewed as a GM. Patients who did not carry the *LAMA5* variant presented only microscopic hematuria. The same authors reported another family with a male proband who had presented with severe microscopic hematuria at 18 months of age. At the age of 13 years, he had low grade proteinuria, 400 mg/24 h, which by the age of 26 years had reached the range of 1.71 g/24 h, for which he was treated with RAAS-inhibitors. A kidney biopsy revealed features of AS that included alternate thinning and thickening of the GBM, along with the fusion of podocytes foot processes. The proband did not exhibit any ocular or audiometric abnormality. Molecular testing revealed a COL4A5:*p*.Gly1107Arg pathogenic variant and LAMA5:*p*.His3130Tyr (Likely Benign, 0.015% in ExAC database). The proband inherited the *COL4A5* variant from the mother, as expected for X-linked inheritance, who only presented with microscopic hematuria, and the *LAMA5* variant from the father who had microscopic hematuria and unilateral hypoacusia.

### 2.4. MYH9 (Non-Muscle Myosin Heavy Chain-9)

Pathogenic variants in this gene cause a spectrum of disorders, known as the May–Hegglin anomaly, Epstein and Fechtner syndrome, and others, inherited in an autosomal dominant mode. It is a pleiotropic condition with symptoms that affect the eyes, ears, kidneys and thrombocytes [56]. In a previous publication, a 6 year-old girl was described who had inherited from her father the pathogenic variant COL4A5:*c*.2605G>A, *p*.Gly869Arg [30]. The father had typical XLAS, and his daughter presented with microscopic hematuria, proteinuria and early sensorineural hearing loss. The authors attributed the unusually early onset of severe symptoms in the girl to one additional heterozygous variant she had inherited from her mother in the *MYH9* gene, MYH9:*c*.4952T>G, *p*.Met1651Arg (variant of uncertain significance, VUS; never reported in databases). The mother also had inner ear deafness. 

### 2.5. HBEGF (Heparin Binding Epidermal Growth Factor)

Although not related to collagen IV nephropathy, another candidate modifier in familial microscopic hematuria was reported by Papagregoriou et al., in the 3′untranslated region of the *HBEGF* gene. This work was based on the hypothesis that polymorphisms in microRNA binding sites (miRSNPs) might act as GMs when co-inherited with a primary glomerulopathy, such as TBMN and/or CFHR5 nephropathy. Previous research work identified SNP C1936T in the 3′UTR of *HBEGF* (rs13385, 3′UTR+1006), which corresponds to the second position of the seed region of miRNA hsa-miR-1207-5p. Functional studies in undifferentiated cultured podocytes demonstrated that in the presence of a mimic for miRNA hsa-miR-1207-5p, there was down-regulation of the *HBEGF* expression, judged by a western blot analysis. This was supported by in cellulo transient transfection experiments using luciferase sensor constructs of both alleles, where the 1936T allele demonstrated abrogation of miRNA binding. Most interestingly, in a cohort of 78 CFHR5/C3 glomerulopathy patients (45M/33F), the 1936T allele was genetically associated with a higher risk for progression to severe kidney disease; such patients were shown to have microscopic hematuria plus added proteinuria >500 mg/day, or even CKD or kidney failure. When the authors tested this same variant against a separate cohort of 103 patients with TBMN, classified as mildly or severely affected, they failed to show an association [31].

### 2.6. MYO1E (Non-Muscle Membrane-Associated Class I Myosin)

*MYO1E* encodes a non-muscle myosin protein located in the cytoplasmic side of the podocyte membrane, which Lennon et al. [32] showed shares common partners in a complex interactome between the collagen IV in the GBM and podocyte cytoskeletal proteins. Lennon et al. reported on a consanguineous Pakistani family with five children. A pathogenic *COL4A5* allele carried two variants in cis, which was inherited by two affected siblings, one male and one female, and demonstrated a XLAS phenotype much worse than expected. The male patient presented with nephrotic syndrome and progressed to kidney failure at the age of three years with characteristic EM features. His older sister, who carried the variant heterozygously, progressed to kidney failure by the age of eight years, which is unusual for the XLAS in females. A Next Generation Sequencing (NGS) analysis revealed that both patients were also homozygous for the *MYO1E* variant *p*.Thr876Arg, and that the girl was also homozygous for a different *MYO1E* variant, *p*.Lys118Glu. Skewed X-inactivation might also have contributed. Disease-causing variants in *MYO1E* are known to cause a rare hereditary recessive form of focal segmental glomerulosclerosis. It might be more appropriate to describe the situation in this family as digenic inheritance, where two separate, rare inherited nephropathies were co-inherited, thereby exacerbating the phenotype, rather than being a case of XLAS co-inheriting a *MYO1E* GM. According to the authors, the variants in both genes summate to explain the phenotype better than each one alone. This might be a situation where the limits between GM and digenic inheritance are blurred.

### 2.7. SYNPO (Synaptopodin)

*SYNPO* encodes a podocyte actin-associated protein with an important role in maintaining the podocyte foot processes. Although no pathogenic variants in the coding region of *SYNPO* have been reported in patients with hereditary focal segmental glomerulosclerosis, there are two variants described in the promoter region of *SYNPO*, upstream of the translation start site, in two patients with focal segmental glomerulosclerosis [57]. Work in mice showed that SYNPO might not be necessary for supporting normal kidney function, but it confers protection to injured podocytes [58]. When *Synpo* and *Col4a5* mutant mice were intercrossed to generate XLAS mouse models lacking synaptopodin, kidney disease was accelerated, with worse outcomes in the following: significantly reduced lifespan, higher levels of albuminuria, glomerulosclerosis and foot process effacement [34]. The authors speculated that this might be related to easier detachment of podocyte foot processes from the GBM and provided evidence of cytoskeletal abnormalities in podocytes. Future studies into a *Synpo*-driven kidney protection mechanism in AS can potentially determine the potential of *Synpo per se* as a target for the development of novel therapeutic approaches or whether alternative targets should be sought regarding the podocyte actin cytoskeleton (Table 2).

### 2.8. Fmn1 (Formin 1)

This gene was shown to be related to albuminuria in humans and in the formation of actin filaments and the microtubule cytoskeleton, modulating the polymerization of linear actin cables. In elegant work in diversity outbred mice, Takemon et al. showed that a variant in *Fmn1*, *p*.Glu377Gly, which evidently affects the proper protein function, was associated with reduced albuminuria in mice with XLAS. After the crossing of relevant strains of mice, they obtained animals that expressed either one or both *Fmn1* null alleles (disrupted exon 9). They showed that in heterozygous females and males, there was a two-fold reduction in Fmn1 protein levels, and they also had a lower albumin to creatinine ratio at 6-weeks of age. Electron microscopy showed that mice that co-inherited the AS phenotype and one dose of the *Fmn1* normal gene had fewer abnormalities with less foot process effacement and fewer protrusions of the podocyte foot processes into the GBM. Therefore, variants abolishing the normal function of this specific gene emerge as having a modifier protective effect [35].

### 2.9. DDR1 (Discoidin Domain Receptor-1)

*DDR1* encodes a collagen receptor tyrosine kinase that can interact with collagen IV as well as with fibrillar collagens that are deposited in fibrotic areas, making it an especially attractive candidate modifier gene in AS. Work originally published by the group of Oliver Gross supports this hypothesis. In the *Col4a3−/−* autosomal recessive model of AS, they also knocked-out the *Ddr1* gene. In doubly knocked-out mice, there was less fibrosis and inflammation, while the mice lived 47% longer compared with AS mice with *Ddr1* expression. Kidney function was significantly improved, as evidenced by reduced proteinuria and blood urea nitrogen. These findings highlighted the importance of crosstalk between the podocytes and the GBM through DDR1 receptors. Based on this, it can be assumed that AS patients, who by serendipity co-inherit a *DDR1* variant, might manifest a less severe phenotype, and one could consider this gene as a probable therapeutic target [36]. Indeed, a small molecule DDR1 inhibitor was found to preserve kidney function and reduce tissue damage in an AS mouse model [59].

Sannomiya et al. [60] hypothesized a similar role for *DDR2*, which is homologous to *DDR1*. They studied mice with the X-linked form of AS and knocked-down *Ddr2* using *Ddr2*-specific allele specific oligonucleotides. This treatment decreased *Ddr2* expression but failed to improve proteinuria or decrease the blood urea nitrogen level, nor did it significantly ameliorate the kidney injury, inflammation or fibrosis in AS mice. They concluded that *DDR2* may not be critically involved in AS pathology [60]. 

All candidate GMs reported here are described in Table 1, and a list of gene knockouts/mutants that have been combined with AS mouse models, in addition to those discussed above, are shown in Table 2.

Finally, it is worth mentioning that the specific strain, and the genetic background on which the collagen IV pathogenic variant is established and transmitted in AS mouse models, is of paramount importance in determining the severity of the final phenotype. It is well known that the same *Col4a* defect on the murine 129X1/SvJ or C3H background manifests a more severe phenotype compared with when it is on the C57BL/6J background [61,62,63] and Deltas C et al., unpublished results]. These observations suggest that many genetic markers are responsible for determining the final phenotype of a specific variant of AS. For a comprehensive review on AS mouse models and their genetic background, see [64].

## 3. Discussion

We described published works advocating for the modifying role of several variants, avoiding being too critical, as most were published by the authors’ groups. Interested researchers can scrutinize the original works. We understand that some of the data are somewhat weak as the shown associations are not based on sound statistical power (except for the *NEPH3* variant) [28] but rather, they represent sporadic incidences with anecdotal evidence in small or larger families. Others are supported by case-control studies with a variable number of participants, familial segregation studies and/or extensive cell culture functional assays. All GMs were identified through a candidate gene approach.

Based on the relevant publications, focal segmental glomerulosclerosis has emerged as a frequent histological finding in kidney biopsy specimens of patients with heterozygous disease-causing *COL4A3/A4* variants, including in patients with AS. This has often resulted in misdiagnosis and the provision of erroneous immunosuppressive medications with anticipated negative repercussions [13,14]. Clinically, these patients present with microscopic hematuria and proteinuria and the first-tier approach for examination should be genetic testing for variants in the genes involved in familial microscopic hematuria [8,9,65]. Elegant work by several authors proposed a mechanism of podocyte detachment and depletion in patients with thin GBM or typical AS, thus leading to proteinuria and focal segmental glomerulosclerosis [66,67,68,69].

One can hypothesize that true recessive pathogenic variants, inherited heterozygously in one of many known genes that are associated with persistent proteinuria, do not confer any clinical symptoms; however, when found on the background of another disease which has a different primary genetic cause, they may exacerbate or aggravate the phenotype, acting as true GMs. In case the second co-inherited variant is in a dominant gene causing proteinuria, with complete penetrance, then we are entitled to invoke true digenic inheritance to better explain the full phenotype. It is interesting that, based on available literature, not a single collagen IV variant has been proposed as a modifier, yet, regarding Alport spectrum diseases. The occasions where two variants were identified, one in each of the *COL4A3/A4/A5* genes, were interpreted as the result of digenic inheritance with a variable severity, generally considered somewhere in between the true typical AS and heterozygous *COL4A3/A4* genotypes [70]. All published modifiers encode podocyte proteins expressed in the glomerular filtration barrier, including the GBM or connected to the cytoskeleton. Amongst the limited number of publications, the *NPHS2* gene (podocin) has been verified by several groups as a genuine candidate modifier, because it is a true recessive gene with which heterozygosity is not known to be associated with any noticeable phenotype. 

Although there is no evidence for its probable causality, one cannot exclude the potential modifier role of the reduced expression of the collagen IV allele that is inherited from the healthy parent. No experimental results are known that support this hypothesis and it is difficult to perform due to the challenge posed by the need for high quality mRNA, preferably from the kidneys of the patients to accurately assess the level of collagen gene expression.

In several cases, the distinction between a true GM effect and digenic inheritance is blurred. In the absence of a patient carrying only the non-collagen IV variant, it is challenging to predict its effect [22,71]. Another way to look at it, which wishfully removes any potential ambiguity, is that the clinical diagnosis by the attending clinician is clearly attributed to a responsible primary DNA pathogenic variant. However, the full spectrum of the disease symptoms behaves as a multifactorial condition, implicating the primary gene, modifier gene(s) and environmental factors [12,72]. The case at hand is familial microscopic hematuria due to heterozygous disease-causing variants in the *COL4A3* or *COL4A4* gene, or even the *COL4A5* gene (in women), responsible for the X-linked form of AS.

The examples so far point to the rarity of strong variant effects, many more of which may exist in the general population. No studies have been able to demonstrate relatively frequent variants that would aggravate the phenotype of a patient with Alport spectrum nephropathy. There are occasions where the second, co-inherited variant might represent a variant responsible for a related but independent phenotype, while in other situations, it is clear that the second variant is evidently totally neutral on its own (e.g., NPHS2:*p*.Arg229Gln). Perhaps it would be preferable to expand the GM definition to include variants that enable us to describe the phenotype of the patient better. These could be heterozygously inherited variants in recessive genes, which on their own confer no perceptible phenotype, or hypomorphic variants that may or may not be accompanied with any symptoms, when inherited singly. Examples of hypomorphic variants could be the ones described in the *LAMA5* and *MYO1E* genes.

Another published example pertains to CFHR5 nephropathy, which although it is not an Alport spectrum disease, follows a clinical course very similar to that in patients with TBMN. The *MYH9* variant rs11089788 (MYH9:c.-19-5801G>T; 45.42% in gnomAD Aggregated database) was shown in one study [33] to confer a higher risk for severe kidney disease in a cohort of patients with CFHR5 nephropathy/C3 glomerulopathy. All patients were heterozygous for an exon 2-3 duplication in the *CFHR5* gene, which encodes a complement activation regulatory protein, highly homologous to the complement factor H (CFH). The same study did not demonstrate a genetic modifier effect in a cohort of TBMN patients [33].

Variants in other genes that are known to have a role in the development and function of podocytes, the GBM and the slit diaphragm, affecting the glomerular filtration barrier, as well as genes regulating inflammatory, fibrotic and ER stress pathways, might be good candidates to look to for potential modifiers. Based on the limited published results, it is evident that many more GMs remain to be discovered. One hopes that some of them may turn to represent pharmacological targets, thereby promoting the discovery of new treatments, either effective enough to stop kidney function decline or at least to delay it significantly, either alone or in combination with approved existing medications.

There are several other monogenic conditions in which the full phenotypic spectrum depends on the contribution of co-inherited GMs. One notable example is cystic fibrosis (*CFTR* gene), a relatively frequent potentially lethal autosomal recessive disease. With a carrier frequency of about 1/25–30 in the Caucasian population, cystic fibrosis is a pleiotropic disease involving several organs, which is recognized to have a polygenic aetiology, to explain its complete phenotypic continuum. Among many suggested GMs, a validated GM is a variant in the *TGFβ1* gene, well-known for its inflammatory and remodeling role, thus exerting an effect on asthma and chronic obstructive pulmonary disease [73].

Finally, it should be mentioned that despite the enthusiasm about the potential role of GMs, other non-genetic factors, including obesity, lifestyle and medication, as well as epigenetic mechanisms, might claim, separately or in combination, a part in determining the clinical phenotype. Future experimental work, supported by deep phenotyping of patients in large cohorts, will enable the unbiased identification of such factors, even of a small effect, on statistical grounds. 

## 4. Conclusions

**Figure 1:**
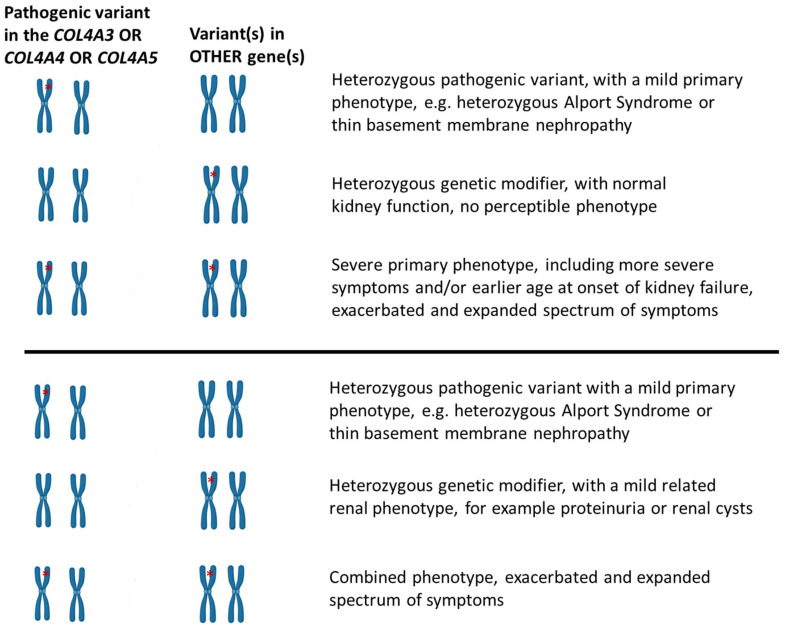
Schematic representation of the hypothesized modifier role of co-inherited DNA variants in patients who present with a more severe phenotype of the collagen IV nephropathy than normally anticipated. (**The upper scenario**) depicts the situation where the putative modifier on its own is absolutely benign, or hypomorphic, not conferring any symptom or disease feature (e.g., heterozygous NPHS2:p.Arg229Gln). (**The lower scenario**) depicts the situation where the putative modifier is accompanied with some features, usually very mild, and together, the primary disease-causing variant and the modifier better explain the clinical picture of the patient than each one alone. It is also understood that more than one candidate for genetic modifiers might co-exist, thereby recapitulating a situation where the full phenotype is the result of oligogenic inheritance. A variation of these scenarios is when the co-inherited genetic modifier is in a homozygous state (see the case of *MYO1E* variants described here) [32]. The red asterisk depicts the genetic variant, either the primary *COL4A* pathogenic variant or the genetic modifier.

It is not always known or recognizable as to how the candidate GMs exert their detrimental effect, although a general scenario is depicted in Figure 1. In some cases, the known function of the affected proteins implies that a long slow process is in place, which accentuates the mutant collagen misfunction, thereby exacerbating the phenotype in terms of severity and age at onset. The variants that act as candidate GMs and described here are in proteins that are part of the GBM or the podocyte cytoskeleton, involved in the essential crosstalk. This crosstalk or interaction is impaired to a small but evidently perceptible level, contributing to the phenotype, most times by predisposing to the development of focal segmental glomerulosclerosis on the long follow-up.

It is worth searching for candidate GMs, both because this approach will enable us to identify more genes that contribute to the disease mechanism, and to detect potential targets for treatment with re-purposed or new medications. As these lines are being written, multiple groups may be applying massive parallel sequencing technologies in search for potential GMs in an unbiased and more promising fashion, using well-studied patient cohorts.

Thus far, the limited list of putative GMs, described by exploring the candidate gene approach (Table 1), does not include a recognizable drug target in humans. Also, it is worth suggesting a search for more frequent modifier variants, which will require large enough cohorts of patients with collagen IV nephropathies, through the exploitation of existing or new registries at a European (for example, through the UK Biobank) or even global level. The use of animal approaches, such as diversity outbred mice [35], are not a robust method to be applied by the average laboratory, especially when there is no easy access to an animal house facility and appropriate funding. In the absence of other functional studies, we could combine cohorts of patients with the required statistical power and resolve to genome wide arrays in search for variants associated with the desired phenotype. Especially with regards to familial microscopic hematuria, a very frequent hereditary feature, this would not be impossible under the interest of the International Alport Syndrome Consortium.

## Figures and Tables

**Table 1:** Putative genetic modifiers published previously in patients with inherited microscopic hematurias, including Alport Syndrome. Variants aggravating CFHR5 nephropathy/C3 glomerulopathy are also included. Although CFHR5 nephropathy is a complement disorder with a different pathomechanism, and is not part of Alport spectrum diseases, it follows a clinical course very similar to thin basement membrane nephropathy. Patients with CFHR5 nephropathy were examined in mixed cohorts with patients inheriting thin basement membrane nephropathy. The references with asterisks (*) also provide cell culture functional data. ^a^ Minor Allele Frequency (MAF) among 1000 Exomes of the Cypriot general population, *CYPROME*.

Primary Gene Defect	Co-Inherited Likely Modifier Gene Variant(s)	MAF (gnomAD)	MAF (CYPROME) ^a^	Comments	Reference
*COL4A3*	*NPHS2*:p.Arg229Gln (rs61747728)	0.03025	0.0166	Tested in a cohort of patients carrying several pathogenic variants and in functional studies in cell culture	[23,24,25,26,27] *
*COL4A3*	*NPHS2*:p.Glu237Gln (rs146906190)	0.0007370	0.0055	Tested in families and functional studies in cell culture	[25] *
*COL4A5*	*NPHS1*:p.Arg408Gln (rs33950747)	0.04859	0.0151	Tested in families	[26]
*COL4A4*:c.1109G>A p.Gly370Glu (rs779604374)	*LAMA5*:c.5149C>T, p.His1717Tyr (rs875379)	0.07186	0.1039	Tested in a family that the authors diagnosed with ADAS	[27]
*COL4A5*:c.3319G>A p.Gly1107Arg (rs104886225)	*LAMA5*:c.9388C>T, p.His3130Tyr (rs201154340)	0.0001200	0.0005	Tested in a family with XLAS	
*COL4A4*:c.4444del p.Leu1482Trpfs*70 (NM_000092.5)	*LAMA5*:c.2321C>T, p.Thr774Ile (rs145721906), co-inherited with *NPHS2*:c.686G>A, p.Arg229Gln (rs61747728)	0.001418 0.03025	0.004 0.016	Tested in a family that the authors diagnosed with ADAS	
*COL4A3* and *COL4A4*	*NEPH3*:p.Val353Met (rs35423326)	0.02863	0.0306	Tested in a large cohort of patients carrying several pathogenic variants and in cohorts of the general population. Also, tested with extensive cell culture assays.	[28] *
*COL4A5*	*LAMA5*:p.Pro1243Leu (rs756101090)	0.00004538	Not detected	Tested in a family segregating XLAS	[29]
*COL4A5*:c.2605G>A, p.Gly869Arg (rs104886189)	*MYH9*:c.4952T>G; p.Met1651Arg (NM_002473.6)	Not detected?	Not detected	Found in one female patient	[30]
*CFHR5*	*HBEGF* (Heparin Binding Epidermal Growth Factor) SNP C1936T in the 3′-UTR of *HBEGF*, 2nd position of seed region of miRNA hsa-miR-1207-5p c.*1006C>T (rs13385) The asterik denotes that the position is 1006 nucleotides past the translation STOP codon	0.1988	Information Not available	Tested in patients with CFHR5 nephropathy/C3 glomerulopathy, due to a founder *CFHR5* exon 2-3 duplication. Also tested in cell culture functional studies.	[31] *
*COL4A5*:c.2858G>T; p.(Gly953Val) (rs78972735) AND in cis c.3097G>C; p.(Gly1033Arg) (NM_033380.3)	*MYO1E*:c.352A>G; p.(Lys118Glu) (NM_004998.4) Homozygous *MYO1E*:c.2627C>G; p.(Thr876Arg) (rs147596471) Homozygous	Not detected?	Not detected 0.0010	Found in a brother and sister in a family segregating XLAS	[32]
*COL4A* and *CFHR5*	*MYH9*:c.-19-5801G>T (rs11089788)	0.4604	Information Not available	Tested in a cohort of patients carrying several *COL4A* variants or the *CFHR5* exon 2-3 duplication	[33]

**Table 2:** Summary of potential genetic modifiers published previously, which report on mouse models with Alport Syndrome, with one additional mutated gene.

Potential Modifier Gene (Knocked-Out or Heterozygous Mutant)	Impact on Kidney Disease Progression	Reference
*SYNPO* (Synaptopodin)	Accelerated kidney disease progression due to podocyte cytoskeletal defects and easier detachment from the GBM	[34]
*Fmn1* (Formin 1)	*Fmn1* heterozygosity reduced albuminuria, podocyte foot process efffacement and podocyte protrusions into the GBM	[35]
*Ddr1* (Discoidin Domain Receptor-1)	Slowed kidney disease progression; extended lifespan	[36]
*Alb* (Albumin)	Reduced glomerular and tubulointerstitial pathology and increased lifespan by 64% on the C57BL/6J background	[37]
*Itga1* (Integrin α1)	Delayed onset of proteinuria and preservation of podocyte foot process architecture. Inhibiting TGF activity or expression synergized with *Itga1* knockout to further slow kidney disease progression.	[38]
*Itga2* (Integrin α2)	Delayed glomerulosclerosis and tubulointerstitial fibrosis, reduced proteinuria, improved GBM architecture, increased lifespan by 20%.	[39]
*Itgb6* (integrin β6)	Decreased renal fibrosis and smooth muscle actin+ cells, consistent with reduced TGFb expression	[40]
*Lamb2* (laminin β2) null and p.S83R mutations	Worsened kidney disease progression	[41]
*Mmp9* (Matrix metalloproteinase 9)	No discernible impact on kidney disease progression or GBM architecture	[42]
*Spp1* (Osteopontin/OPN)	Reduced proteinuria, high blood pressure and GBM thickening and increaased lifespan	[43]
*Trp53* (p53); podocyte-specific mutation	Increased foot process effacement and renal dysfunction	[44]
*Usag1* (Uterine-sensitization-associated gene 1)	Attenuation of disease progression, normalization of GBM ultrastructure, preservation of renal function and extension of life span	[45]

## Data Availability

Data is available through the original publications.
